# Case fatality rate and associated factors of mortality among first-ever stroke patients: a prospective cohort study in Erbil City, Iraq

**DOI:** 10.11604/pamj.2025.52.139.49340

**Published:** 2025-12-04

**Authors:** Qasim Abbood Kareem, Kameran Hassan Ismail

**Affiliations:** 1 Department of Community Medicine, College of Medicine, Hawler Medical University, Erbil, Iraq

**Keywords:** Stroke, mortality, risk factors, heart, ischemic

## Abstract

**Introduction:**

stroke is the second most common cause of death worldwide, with ischemic heart disease exceeding it. This study aimed to identify the hospital-based case-fatality rate and its associated factors in Erbil city.

**Methods:**

a hospital-based prospective cohort study was conducted on 118 first-ever stroke patients who were diagnosed by a consultant internist or neurologist and confirmed by brain Computed Tomography (CT) or Magnetic Resonance Imaging (MRI), admitted at Erbil teaching hospitals from 20^th^ January to 20^th^ May 2025. Data were collected in two stages: the first was during the onset of the stroke by interviewing the patients or their close relatives, and the second was after a month of follow-up through interviews or telephone.

**Results:**

these results showed that out of 118 patients with stroke, the one-month hospitalized stroke case fatality rate was 26.3% (95% confidence interval: 18.6% -35.2%). Univariate Cox regression analysis showed that patients older than 65 years had a significantly higher risk of death (HR = 4.349; 95% CI: 1.782-10.610, p = 0.001). Furthermore, stroke patients who had a history of hypertension, diabetes mellitus, heart disease, and dyslipidaemia tended to have a higher risk of death (HR: 3.052, 2.172, 3.477, and 2.295, respectively).

**Conclusion:**

the hospital-based case-fatality rate was relatively high in the first weeks after a first-ever stroke. The early mortality was affected by several factors, including older age, hypertension, diabetes mellitus, heart disease, dyslipidemia, and hemorrhage subtype. It is advisable to strengthen follow-up procedures and maximize guidance for patients' families after their discharge from the hospital.

## Introduction

A stroke is a neurological condition that is distinguished by the obstruction of blood vessels. Blood clots develop in the brain, which disrupts blood flow, obstructs arteries, and causes blood vessels to rupture, resulting in hemorrhage. The sudden mortality of brain cells as a result of a lack of oxygen is the consequence of the rupture of the arteries that supply the brain during a stroke. Depression and dementia may also result from stroke [1]. Cognitive impairment, limitations in daily living activities, and a diminished quality of life are frequently observed as the consequences of stroke [2]. It is the second most common cause of death worldwide, with ischemic heart disease exceeding it [3]. The World Stroke Organization reports that every year, more than 13.7 million stroke attacks occur, with 60% of these cases occurring in individuals under the age of 70 [3,4]. The lifetime risk of having a stroke in people who are 25 years and older is 24.9% [4]. Ischaemic stroke attacks result in the deaths of over 2.7 million individuals annually [4]. Brain damage may result from the rupture of a cerebral artery (hemorrhagic stroke) or blockage (ischaemic stroke). Acute ischaemic stroke (AIS) is the most prevalent type of stroke, accounting for approximately 85% of clinical cases [4]. Cardiovascular diseases, including atrial fibrillation or atherosclerosis of the main arteries, are the most prevalent causes of thromboembolism [3]. Previous studies in Erbil hospitals indicate that the mortality rate among stroke patients was relatively high, reaching approximately one-quarter of all hospitalized cases, and that it increased among patients with older age and gender, as well as other factors such as smoking and cardiovascular disease [5]. In high-income countries, the incidence and mortality rates of first stroke cases have declined gradually in recent decades. However, in numerous low and middle-income countries, these rates have either remained constant or have increased [6].

Studies on non-fatal stroke have shown that as many as two-thirds of stroke patients subsequently die from cerebrovascular disease or ischemic heart disease. Following vascular disease, cancer is the second-leading cause of death among stroke survivors and thus contributes to approximately 12% of all-cause mortality, while infections, trauma, and other causes make up the remainder [7,8]. Stroke mortality rates have been decreasing in nearly every nation [9]. A decrease in mortality may be the consequence of a decrease in the occurrence of the disease, a decrease in the number of cases, or both. Improved risk factor management, which can be accomplished through lifestyle modification and prevention, may lead to a decrease in stroke event rates. People who survive a stroke have a high rate of disability and an increased risk of developing vascular dementia, which is why a decline in disease is preferable to a decline in case fatality from a public health perspective [10]. Nevertheless, the quality of care is of the utmost importance to the survival of stroke patients and their families. The decreased case fatality at 30 days following a stroke is probably a consequence of advancements in treatment and management, and potentially in prevention, which could mitigate the severity of strokes [11]. Stroke is the most common cause of death and disability globally, especially in underdeveloped nations like Iraq, where short-term outcomes data are scarce. The case fatality rate (CFR) indicates acute stroke treatment quality and early management and rehabilitation effectiveness. However, Erbil City has little local data on first-stroke mortality and its causes. Identifying age, gender, behavioural characteristics, medical history, and stroke subtype as risk factors for early stroke mortality can inform prevention and treatment. Thus, this prospective cohort study estimated the hospital-based case fatality rate and identified the primary determinants of mortality among first-ever stroke patients in Erbil after 2018, providing vital data for improving stroke outcomes and healthcare planning in Iraq. Therefore, this study aimed to identify the hospital-based case-fatality rate and its associated factors in Erbil city, Iraq. It was hypothesized that the case fatality rate would be higher among older patients, those with haemorrhagic stroke, medical history such as hypertension, heart diseases, dyslipidaemia, and diabetes.

## Methods

**Study design:** a hospital-based prospective cohort study.

**Setting and time of the study:** this study was conducted at Rizgary and Hawler teaching hospitals in Erbil from January 20^th^ to May 20^th^, 2025. During this period, all eligible first-time stroke patients were consecutively recruited. The patients or their close relatives were interviewed directly during hospital admission using a structured questionnaire to collect baseline information. A one-month follow-up was conducted on each patient from the date of admission to ascertain their survival status. Following up was implemented through either a subsequent home visit or a scheduled phone call. A full 30-day observation period was guaranteed for all participants, as the final patient follow-up was conducted on June 19, 2025.

**The study population:** this study included 118 first-ever stroke patients hospitalized, who were selected by a convenience sampling method. Cases involving the first stroke attack (first-ever stroke) were diagnosed by a consultant internist or neurologist and subsequently confirmed by a brain Computed Tomography (CT) or Magnetic Resonance Imaging (MRI). Patients were followed up for one month to measure the hospital-based case-fatality rate.

**Inclusion and exclusion criteria:** this study included males and females aged 18 years or older who were residing in Erbil city. It excluded patients with transient ischemic stroke (TIA), brain metastasis, brain tumour, space-occupying lesions, or those caused by trauma and other stroke mimics.

**Sample size:** according to Daniel and Cross's (2018) [12], formula for calculating sample size was used to calculate the sample size. The following information was entered into formula: Expected rate (P) of stroke based on the survey in the Middle East, including Iraq, the incidence rate for stroke was 0.25% [13]. With an absolute precision (d 2) of 1%. With Z-score corresponding to the level of confidence (for a 95% confidence level, Z ≈ 1.96). Since the sample size (n) was 96, taking into account the possibility of missing follow-up data and missing data, the target sample size was increased by 12%, resulting in a final sample of approximately 118 patients. This ensured sufficient statistical power (=80%) to detect statistically significant associations between demographic data and predictors and mortality outcomes.


$$n=\frac{z^{2}P\left(1-P\right)}{d^{2}}$$

**Data collection method:** data were collected through in-person or telephone interviews with patients or their relatives, or were recorded from hospital records one month after the interview date, using a questionnaire designed by the researchers. Data required included age and sex. Smoking status was also required to determine whether the patient was a current smoker, ex-smoker, or non-smoker. Current smoking is defined as continuous or cumulative smoking for ≥ 6 months in a person's life, smoking at least one cigarette per day before the time of stroke [14,15]. Ex-smokers are defined as people who have stopped smoking whether for less or more than 5 years at the time of the interview [16]. Alcohol consumption is defined as consuming at least one drink per week for a minimum period of 6 months before the stroke [17,18]. Exercise was assessed based on a self-report on the frequency of exercise over the previous year before the stroke date (cases). Exercise was categorized as "yes" (walking at least 30 minutes per day) or "no" (walking less than 30 minutes per day or not at all) [19,20]. Medical history, such as hypertension, diabetes mellitus, heart diseases, dyslipidemia, and depression, was defined as conditions self-reported by participants as having been previously diagnosed by a physician or using condition-related medications before the stroke [21,22]. Family history of stroke was defined by self-report as ≥ 1 first-degree relative with stroke [23]. Stroke subtypes were classified as ischemic and hemorrhagic based on the physician's diagnosis documented in the patient's file.

**Questionnaire's validity:** the validity of the content of the questionnaire was confirmed by showing it to a group of eight experts. These experts were requested to evaluate the questionnaire's clarity, relevance, and suitability for achieving the current study's objectives. They suggested implementing minimal modifications.

### Questionnaire's reliability

Reliability concerns the extent to which a measurement of a phenomenon provides stable and consistent results. Reliability is also concerned with repeatability. Their reliability was assessed by test-retest. Cohen's kappa is a statistic that is used to measure inter-rater or test-retest reliability for qualitative (categorical) items. It is generally thought to be a more robust measure than a simple percent agreement calculation, as Κ incorporates the possibility of the agreement occurring by chance. At the start of the study, 8 cases were included. These samples were taken, and telephone numbers were recorded upon enrollment. The questionnaire was readministered by telephone or home visit 7 days later. Cohen's kappa coefficient was calculated as appropriate for the item type. The measure of agreement of factors ranged from 0.714 (substantial agreement) to 1.000 (perfect agreement).

**Ethical considerations:** ethical approval was obtained from the scientific and ethical committee of the College of Medicine/Hawler Medical University, the Erbil Health Directorate in Erbil City, and the participants' verbal agreement before the interview. The researcher informed participants or close relatives that the data would be kept confidential and anonymous and that the tool did not contain sensitive personal information.

### Statistical analysis

Before the analysis, the data were checked for missing values, outliers, and inconsistencies. And all study variables were checked for normality using QQ plot, Shapiro-Wilk, and Kolmogorov-Smirnov tests of normality. The data were entered into a personal computer and analyzed using Statistical Package for the Social Sciences-27 (SPSS-27). Code forms were generated for each questionnaire answer. Simple statistics, such as the mean, standard error, frequency, and percentages, were calculated. Fisher's exact (when the expected frequencies of each cell of the table was less than 5) or Chi-square tests were used to compare proportions between independent variables (age, gender, behavioral characteristics, medical history, and stroke subtypes) and the hospital-based case-fatality rate.

Before regression analysis, the goodness-of-fit test was conducted. Multicollinearity diagnostics were performed before running the multivariate logistic regression model. Pearson's correlation coefficients among independent variables were all below 0.8. Variance Inflation Factor (VIF) values were < 5, and tolerance values were > 0.2, indicating no significant multicollinearity. The model showed a good overall performance. The Omnibus Tests of Model Coefficients indicated that the logistic regression model was statistically significant compared to the null model (χ² = 43.804, df = 11, p < 0.001), suggesting that the predictors included in the model significantly improved the prediction of mortality among first-ever stroke patients. Univariate Cox regression analysis to identify factors associated with stroke mortality during the 30-day follow-up period for each patient. In the multivariate Cox proportional hazards regression model, variables were selected based on their significance in univariate analysis (p < 0.05). The Kaplan-Meier/Nelson-Allen approach was used to identify the survival function by subtypes of stroke and time of follow-up. Means for survival time by Log Rank (Mantel-Cox). The P-value was considered statistically significant when it was equal to or less than 0.05.

## Results

**Figure 1:**
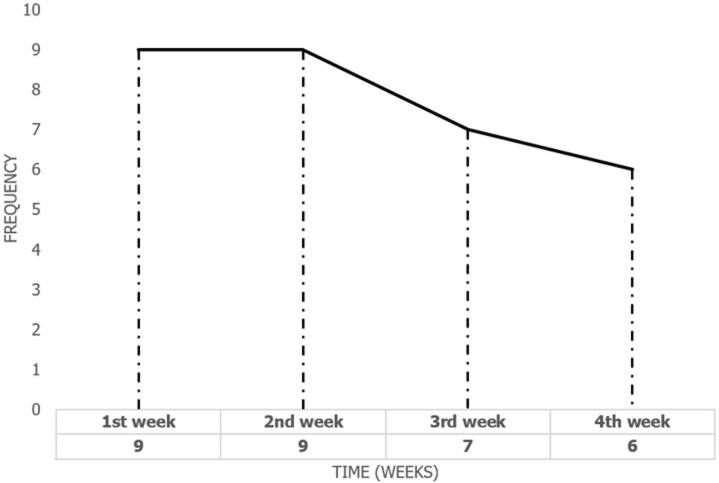
frequency of stroke deaths by week in the 30-day follow-up period (N=31 deaths) in Erbil hospitals

A total of 141 suspected stroke patients were screened for eligibility during the study period. Of these, 23 patients were excluded due to a previous history of stroke or were aged less than 18 years. The remaining 118 patients met the inclusion criteria and were enrolled in the study without loss of follow-up. The present results showed that out of 118 patients with stroke, the one-month case fatality rate of stroke among hospitalized patients was 31 (26.3%) with a confidence interval (95% CI; 18.6%-35.2%). Furthermore, the results indicated that the hospital-based case fatality rate in stroke patients increased to 9 (29.0%) deaths in the first and second weeks. While the case fatality rate dropped to only 7 (22.6%) deaths in the third week, and dropped to 6 (19.4%) deaths in the fourth week as illustrated in Figure 1.

There was a statistically significant association between the hospital-based case fatality rate and variables such as age, hypertension, diabetes, heart disease, dyslipidemia, and stroke subtypes (P < 0.05). This shows that older age, those with high blood pressure, diabetes, heart disease, dyslipidemia, and hemorrhagic stroke were associated with an increased stroke mortality rate, as shown in Table 1. Univariate Cox regression analysis based on time-to-death showed that patients older than 70 years had a significantly higher mortality risk during the follow-up period; the hazard ratio was 2.895 (95% CI: 1.404-5.969, p = 0.004). Similarly, stroke patients who had a history of hypertension, diabetes mellitus, heart disease, and dyslipidaemia tended to have a higher risk of death during follow-up (hazard ratios: 3.052, 2.172, 3.477, and 2.295, respectively). However, multivariate Cox regression analysis revealed that patients over 70 had a substantially greater likelihood of dying during the follow-up period than patients under 70 (HR = 2.866; 95% CI: 1.374-5.977, p = 0.005). Similarly, stroke patients with a history of heart disease and hypertension were more likely to die during follow-up (HR = 2.781; 95% CI: 1.322-5.851, p = 0.007) and (HR = 2.596; 95% CI: 1.263-5.339, p = 0.009), respectively. No statistically significant effects were found for other variables, as shown in Table 2.

**Table 1:** case-fatality rate by age, gender, behavioral characteristic, and medical history

Total No. of cases (118)		No. of cases	Dead	Alive	P-value
			Freq. (%)	Freq. (%)	
**Age groups**	< 50 years	7	1 (14.3)	6 (85.7)	0.049**
	50-59 years	22	4 (18.2)	18 (81.8)	
	60-69 years	42	7 (16.7)	35 (83.3)	
	70-79 years	38	14 (36.8)	24 (63.2)	
	≥ 80 years	9	5 (55.6)	4 (44.4)	
**Gender**	Male	58	15 (25.9)	43 (74.1)	0.921*
	Female	60	16 (26.7)	44 (73.3)	
**Cigarette smoking**	Current smoker	43	11 (25.6)	32 (74.4)	0.999**
	Quit ≤5 years	4	1 (25.0)	3 (75.0)	
	Quit >5 years	2	0 (0.0)	2 (100.0)	
	Never smoker	69	19 (27.5)	50 (72.5)	
**Alcohol drinking**	Yes	22	5 (22.7)	17 (77.3)	0.675*
	No	96	26 (27.1)	70 (72.9)	
**Exercise**	Yes	14	5 (35.7)	9 (64.3)	0.517*
	No	104	26 (25.0)	78 (75.0)	
**Hypertension**	Yes	30	14 (46.7)	16 (53.3)	0.003*
	No	88	17 (19.3)	71 (80.7)	
**Diabetes mellitus**	Yes	63	22 (34.9)	41 (65.1)	0.022*
	No	55	9 (16.4)	46 (83.6)	
**Heart diseases**	Yes	41	19 (46.3)	22 (53.7)	<0.001*
	No	77	12 (15.6)	65 (84.4)	
**Dyslipidaemia**	Yes	32	13 (40.6)	19 (59.4)	0.031*
	No	86	18 (20.9)	68 (79.1)	
**Depression**	Yes	10	5 (50.0)	5 (50.0)	0.125*
	No	108	26 (24.1)	82 (75.9)	
**Family history of stroke**	Yes	38	6 (15.8)	32 (84.2)	0.075*
	No	80	25 (31.3)	55 (68.8)	
**Subtype of Stroke**	Ischemic	99	22 (22.2)	77 (77.8)	0.043*
	Haemorrhage	19	9 (47.4)	10 (52.6)	

p-values are based on Pearson' Chi-squared (*) or Fisher exact test (**) for categorical variables.

**Table 2:** univariate and multivariate Cox regression analysis to identify factors associated with stroke mortality during the 30-day follow-up period

Crude Cox Regression				Adjusted Cox Regression		
Variables	P- value	Hazard Ratio (HR)	95.0% CI for HR	P- value	Adjusted Hazard Ratio (aHR)	95.0% CI for aHR
			Lower-Upper			Lower-Upper
Age (≥70 years)	**0.004**	2.895	1.404-5.969	**0.005**	2.866	1.374-5.977
Gender (female)	0.860	1.065	0.527-2.155			
Smoking (Yes)	0.664	0.852	0.413-1.755			
Alcohol (Yes)	0.649	0.800	0.307-2.085			
Exercise (No)	0.516	0.728	0.280-1.896			
Hypertension (Yes)	**0.002**	3.052	1.503- 6.197	**0.009**	2.596	1.263-5.339
Diabetes mellitus (Yes)	**0.050**	2.172	1.000- 4.720	0.230	1.676	0.721-3.893
Heart diseases (Yes)	**0.001**	3.477	1.685- 7.174	**0.007**	2.781	1.322-5.851
Dyslipidaemia	**0.023**	2.295	1.122- 4.691	0.091	1.946	0.899-4.212
Depression (Yes)	0.088	2.301	0.883- 5.996			
Family history of stroke (Yes)	0.101	0.475	0.195-1.158			

**Figure 2:**
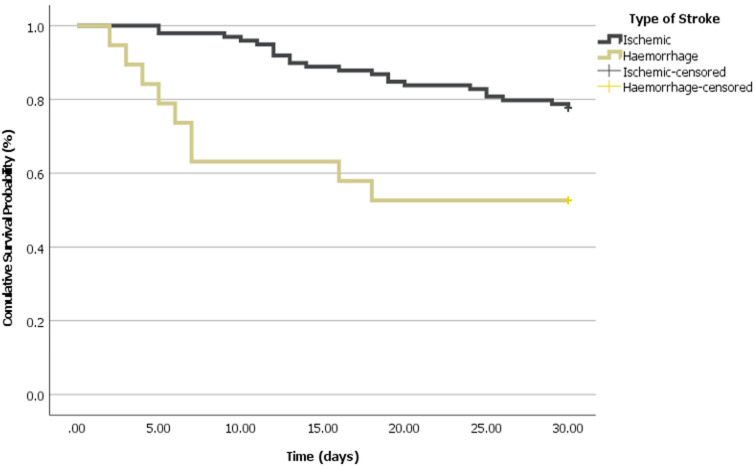
survival function by type of stroke

The analysis in this study indicated that the mean survival time for patients with ischemic stroke was significantly longer, estimated at 27.04 days (SE = 0.668, 95% CI: 25.731-28.350), compared to patients with hemorrhagic stroke whose mean survival time was only 19.37 days (SE = 2.704, 95% CI: 14.068-24.669). The overall mean survival time for all patients was 25.81 days (SE = 0.759, 95% CI: 24.318-27.292). The Log Rank (Mantel-Cox) test indicated a statistically significant difference between the two groups (Chi-Square = 8.540, P = 0.003), suggesting that patients with ischemic stroke had a significantly longer survival period compared to those with hemorrhagic stroke. It should be noted that the estimation is limited to the largest observed survival time if it is censored as seen in Table 3. The Kaplan-Meier survival analysis demonstrates a clear separation between ischemic and hemorrhagic stroke patients. Individuals with ischemic stroke exhibited a consistently higher cumulative survival probability over time compared to those with hemorrhagic stroke, who showed a more rapid decline in survival, as illustrated in Figure 2.

**Table 3:** means for survival time by stroke subtypes

Subtypes of Stroke	Mean^α^			
	Estimate (per days)	Std. Error	95% Confidence Interval	
			Lower Bound	Upper Bound
Ischemic	27.040	0.668	25.731	28.350
Haemorrhage	19.368	2.704	14.068	24.669
Overall	25.805	0.759	24.318	27.292
**Log Rank (Mantel-Cox)/ Chi-Square (8.540), P-value=0.003**				

α. Estimation is limited to the largest survival time if it is censored

## Discussion

### One month-case fatality of the hospitalized stroke

The results indicated that the one-month case-fatality rate from stroke among hospitalized patients was 31 cases (26.3%) with a confidence interval (95% CI; 18.6%-35.2%). These results were consistent with the study findings conducted in Erbil city [24], which discovered that the one-month case fatality rate among hospitalized stroke patients was 28.3%. The 30-day stroke case fatality rate was almost similar to that reported in Iraq, such as in Erbil, Iraq (23.7%) [25], and in Basra, Iraq (22.7%) [26]. Furthermore, another study indicated that the mortality rate was highest (over 20%) in Turkey, Lithuania, and Latvia, and lowest (under 8%) in Korea, the Netherlands, and Norway [27]. The thirty-day mortality rate of ischemic stroke has been estimated at around 15% in high-income countries [11,28]. Additionally, the in-hospital mortality rate is reported to be as high as 3% to 18%, which represents a substantial proportion of all patients [29]. The case fatality rate for one month after a stroke varies from country to country and may be based on the heavy burden of several risk factors, such as older age, lower socioeconomic status, high blood pressure, diabetes, heart disease, dyslipidemia, and the type of stroke. In addition to the poor use of evidence-based secondary prevention.

The highest mortality rate was among hospitalized stroke patients followed during the first and second weeks. Results of this study agreed with Admas *et al*. [30] that the cumulative in-hospital mortality rate of stroke was higher in the first and second weeks of follow-up, compared to that in the third week and the end of the follow-up week. A comparable investigation demonstrated a substantial mortality rate within 30 days of the first stroke episode, with the greatest percentage of individuals passing away within 7 days of hospitalization [31]. This can explain that the acute phase of the disease, when patients are most susceptible to severe neurological damage and life-threatening complications like elevated intracranial pressure, aspiration pneumonia, cardiac arrhythmias, and thromboembolic events, is responsible for the highest mortality rate among stroke patients during the early weeks [32-34]. Another explanation is that the hospital-based case fatality rate is inflated, as more severe cases are admitted to the hospital.

### Associated factors of stroke mortality

The univariate and multivariate Cox regression analysis showed that patients older than 70 years had a considerably greater mortality risk from stroke during the follow-up period. This aligned with the recent studies [31,35-37], which reported that stroke patients who were older age in univariate and multivariate Cox regression analysis were at significantly higher risk of death after stroke. The possible explanation may be that older age is linked to a greater load of comorbid conditions like diabetes, heart disease, and hypertension, as well as a higher susceptibility to problems like infections and decreased mobility [38,39]. Furthermore, elderly patients frequently experience more severe strokes because of delayed healing capacity, reduced collateral circulation, and cumulative vascular injury, all of which lead to worse outcomes and a greater death rate than in younger patients [40-42]. The univariate and multivariate Cox regression analysis showed that stroke patients with hypertension and heart disease had a greater risk of death during follow-up. These results were similar to the previous studies [34,37,43], which found that hypertension and heart diseases independently increased the risk of death from stroke. The possible explanation of these results may be that these comorbidities aggravate the cardiovascular load, lower cerebral perfusion, and raise the risk of consequences such as heart failure, arrhythmias, and subsequent bleeding [44,45]. While chronic hypertension impairs brain perfusion and stroke recovery by causing long-term vascular damage, such as arterial stiffness, endothelial dysfunction, and an increased atherosclerotic load [46]. So, all these reasons may make heart disease and high blood pressure independent factors associated with mortality after a stroke. The univariate analysis results indicated that the risk of mortality was associated with a history of diabetes and dyslipidemia. These results agreed with the studies [35,36], which found that a history of diabetes was related to the hazard of death after stroke. Furthermore, our results aligned with previous research [32], which reported that dyslipidemia was linked to the hazard of stroke mortality. This can explain that chronic hyperglycemia puts patients at risk for cardiovascular problems, stroke, and multi-organ dysfunction by causing arterial damage, decreased endothelial function, and accelerated atherosclerosis [47]. Diabetes also impairs wound healing and the immune system, making a person more vulnerable to infections and having a worse prognosis [48]. Furthermore, dyslipidaemia speeds up vascular damage and restricts blood flow to critical organs, which raises morbidity and mortality [49]. Because of these cumulative consequences, people with diabetes and dyslipidaemia are more likely to experience serious complications and, as a result, have a greater risk of dying than people without the disease.

In this study, patients with ischemic stroke exhibited a consistently higher cumulative survival probability over time compared to those with hemorrhagic stroke. These outcomes agreed with the study findings done in Diyala [50], which found that death from haemorrhage is higher than ischemic death. Also, these findings were consistent with findings reported by another study [36]. The high mortality rate observed within the early duration after the first-ever stroke may be directly due to the effect of neurological damage occasioned by raised intracranial pressure [34]. The research reported that there were no significant associations between variables such as gender, smoking, alcohol consumption, and family history of stroke with mortality. There may be various reasons why gender, smoking, alcohol intake, and family history of stroke do not affect mortality. Most patients had identical in-hospital care and secondary prevention efforts, which may have mitigated these background variables' short-term effects. In the first month after commencement, these factors may affect stroke risk more than post-stroke mortality.

This study may have various limitations. As hospital-based, the data may not be typical of all stroke cases in the community, especially mild strokes that do not reach health care facilities, which may contribute to selection bias and overestimate case fatality rates. Referral bias may occur when teaching hospitals admit more severe patients. This study is largely limited to self-reports from patients or their relatives about historical medical conditions such as hypertension, diabetes, heart disease, dyslipidemia, depression, and family history of stroke. When patients were hospitalized urgently or lacked medical paperwork, hospital medical records, medicine prescriptions, and diagnostic test results were not always available for verification. Due to limited data and the acute nature of stroke hospitalization, this technique was required, but it may have caused recollection bias or disease reporting errors. Despite its drawbacks, hospital-based stroke studies, especially in low-resource countries, use self-reported information to get insight into patients' medical histories. Furthermore, behavioral characteristics including smoking, alcohol drinking, and exercise) were self-reported by the patient or a close relative. This technique may have created information bias since families may not properly recollect the patient's health-related activities, misclassifying exposure factors, and affecting case fatality relationships. Due to the acute nature of stroke presentation and the often absence of comprehensive medical records or diagnostic data, this strategy was required. In resource-limited hospital-based investigations, self- or proxy-reported information is frequent and practical. Also, the one-month follow-up may not reflect stroke patients' long-term mortality. Due to resource restrictions, patient monitoring issues after discharge, and the necessity to focus on early mortality, which is often the most critical phase after a stroke, this short length was chosen. Since recruiting consecutive hospital admissions was easier within the study timeframe, convenience sampling was adopted, but this may restrict the generalizability of the findings.

## Conclusion

The study concluded that the hospital-based case-fatality rate was relatively high in the first weeks after a first-ever stroke. The early mortality was affected by several factors, including older age, hypertension, diabetes mellitus, heart disease, dyslipidemia, and hemorrhage subtype. It is advisable to strengthen follow-up procedures and maximize guidance for patients' families after their discharge from the hospital.

### What is known about this topic

- The systematic diseases such as hypertension, diabetes mellitus, heart diseases, dyslipidaemia, and are reported to be risks of mortality;
- The first weeks of the follow-up were associated with mortality due to haemorrhage stroke subtype.

### What this study adds

- The study added that a history of hypertension, and heart diseases were independent risk factors for stroke mortality.
